# Development and Validation of a Nomogram for Axillary Lymph Node Metastasis Risk in Breast Cancer

**DOI:** 10.7150/jca.100651

**Published:** 2024-10-07

**Authors:** Shijing Wang, He Zhang, Xin Wang, Juanhan Yu, Qingfu Zhang, Yiwen Zheng, Tangbo Zhang, Xiaoyun Mao

**Affiliations:** 1 Department of Breast Surgery, The First Affiliated Hospital of China Medical University, Shenyang, Liaoning Province, 110001, China.; 2 Department of Medical Imaging, Affiliated Hospital of Nantong University, Nantong, Jiangsu Province, 226001, China.; 3 Department of Radiology, The First Affiliated Hospital of China Medical University, Shenyang, Liaoning Province, 110001, China.; 4 Department of Pathology, The First Affiliated Hospital of China Medical University, Shenyang, Liaoning Province, 110001, China.

**Keywords:** Breast cancer, Axillary lymph node, Metastasis, HRCT, Nomogram

## Abstract

**Purpose:** Preoperative assessment of axillary lymph node (ALN) status is essential for breast cancer treatment planning. This study prospectively analyzed risk factors for ALN metastasis by comparing high-resolution computed tomography (HRCT) imaging with pathology and developed a nomogram to aid in diagnosis.

**Methods:** From April 2023 to May 2024, breast cancer patients confirmed by pathology participated in the study. All had chest HRCT before surgery, and ALN samples were anatomically matched to HRCT imaging and pathology. The least absolute shrinkage and selection operator (LASSO) regression helped refine metastasis features, and a nomogram was constructed using the final selected features determined by multivariate logistic regression. The nomogram's performance was evaluated with concordance index (C-index), calibration plot, and decision curve analysis, with internal validation through bootstrapping.

**Results:** A total of 302 ALN from 98 patients were included in this study. The predictors included in the nomogram encompassed the mean CT value, short diameter, border, and shape of ALN, as well as the Ki-67 status and histological grade of the primary tumor. The model exhibited satisfactory discrimination, with a C-index of 0.869 (95% CI: 0.826-0.912) and an AUC of 0.862 (95% CI, 0.815-0.909). The calibration curve demonstrated a high degree of concordance between the predicted and actual probabilities. The decision curve analysis demonstrated that the nomogram was clinically useful when the threshold for intervention was set at the metastasis possibility range of 1% to 86%.

**Conclusion:** The nomogram combined with preoperative pathology and HRCT imaging have the potential to improve the evaluation of ALN status.

## Introduction

Breast cancer is the most prevalent and the most lethal form of cancer among women globally. The most recent global cancer burden data for 2022, released by the International Agency for Research on Cancer (IARC) of the World Health Organization, indicates that there were more than 2.3 million new cases of breast cancer and 670,000 deaths worldwide in 2022^1^. Status of axillary lymph node (ALN) is of considerable consequence for the staging, treatment planning, and prognostic assessment^2^.

Pathological examination remains the most accurate method of assessing ALN status, it necessitates invasive procedures, which inevitably entails some risk^3^. Additionally, non-metastasis lymph node (NMLN) may be misclassified during surgery, leading to unnecessary dissections that don't benefit the patient and may cause complications. Furthermore, studies indicated that maintaining the integrity of NMLN can be advantageous for immunotherapy^4^,^5^. Consequently, there is a pressing clinical requirement for precise and non-invasive preoperative assessment method. Ultrasonography, while cost-effective and versatile, is heavily reliant on operator expertise and equipment, introducing subjectivity^6^. A limitation is its inability to differentiate between localized and advanced ALN metastasis when results are positive^7^. Additionally, detecting deep-seated or bone-adjacent ALN can be challenging due to anatomical constraints. Magnetic resonance imaging (MRI) offers superior soft-tissue resolution and can distinguish metastasis lymph node (MLN) from NMLN with contrast enhancement^8^. However, preoperative MRI is primarily employed to identify occult breast tumor and to plan breast-conserving surgery due to factors such as cost, examination time, and accessibility. Additionally, MRI has limited ability to display the entire axilla, and artifacts caused by cardiac motion can impede the detection of deep ALN^9^.

Computed Tomography (CT) is commonly used to evaluate the progression and distant spread of breast cancer^10^. As the scan covers the axillary region, chest CT provides crucial information about ALN without exposing the patient to additional radiation. Furthermore, chest CT not only reveals deep ALN and Rotter's nodes but also offers a comprehensive view of both axillae, facilitating comparative analysis between the affected and unaffected sides^11^. Previous studies have indicated that standard CT scans based on size criteria can assist in preoperative ALN status assessment and enhance the accuracy of sentinel lymph node biopsy^12^-^14^. However, conventional CT has limitations in depicting small lymph nodes due to scan slice thickness and reconstruction algorithms^15^. Given the lack of pathology for every ALN, the majority of studies have employed an indirect approach to assess ALN status by sampling the entire specimen.

We prospectively integrated pathology with imaging by utilizing the high spatial resolution of HRCT technology combined with spiral CT. A comparative analysis was conducted on an individual ALN basis to develop a nomogram for predicting ALN status, and its performance was comprehensively evaluated.

## Patients and methods

This study was approved by the Medical Ethics Committee of the First Affiliated Hospital of China Medical University (IRB-2024-2023-514). All patients provided informed consent. All methods were performed in accordance with the Declaration of Helsinki and relevant guidelines. All data generated or analyzed during this study are included in this article. No large language models were used in the preparation of this article.

### Patients

From April 2023 to May 2024, 98 patients with breast cancer diagnosed via core needle biopsy (CNB) were prospectively enrolled. The inclusion criteria for the study were as follows: (1) complete pathology and HRCT imaging; (2) the HRCT examination is performed before CNB to avoid any impact on the imaging signs of the lymph nodes caused by the CNB procedure; (3) the interval between preoperative HRCT examination and surgery was less than one week, and no local or systemic treatment was received during this period. The exclusion criteria were as follows: (1) axillary fat or vascular artifacts that affected the assessment of ALN. (2) no ALN on preoperative HRCT imaging.

### Image acquisition

All patients were scanned using a Siemens 64-slice 128-layer spiral CT machine in a supine position with arms raised, either head-first or feet-first, while maintaining a state of respiratory arrest at the end of inhalation. The scanning range extended from the lung apex to the diaphragm. The CT scanning parameters were set as follows: tube voltage of 120 kVp, a matrix of 512, a scanning slice thickness of 1 mm, and 1 mm thin-slice reconstruction. Upon completion of the scan, image reconstruction was performed using a high spatial frequency algorithm. Subsequently, the thin-slice reconstructed images were archived and transferred to the Picture Archive and Communication System (PACS).

### Imaging-pathology comparison

**Figure 1:**
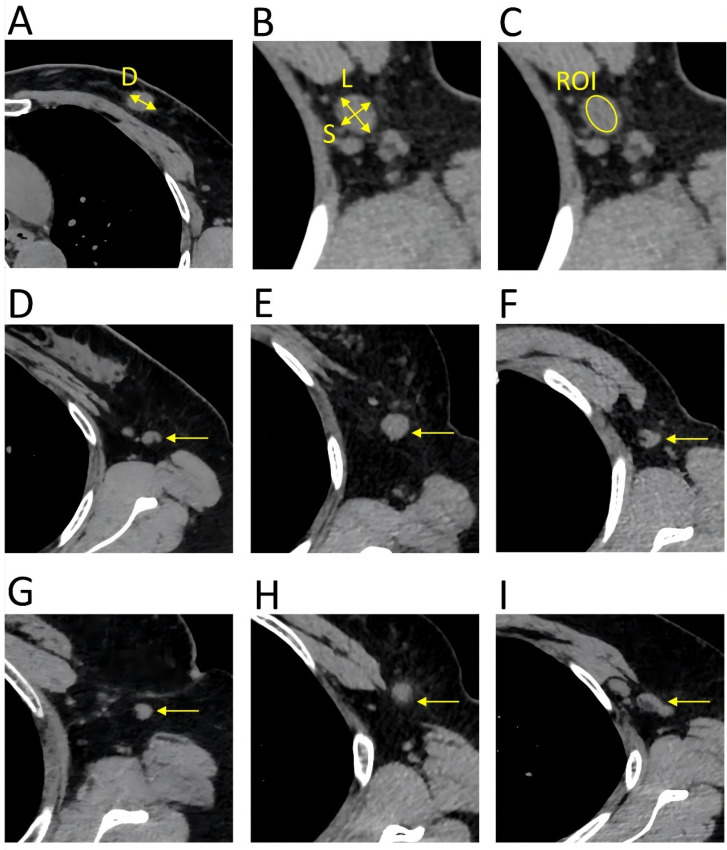
Key parameters of the HRCT imaging. The primary tumor and lymph nodes were evaluated in the transverse of the HRCT imaging, with a slice thickness of 1 mm. A. (D) diameter of the primary tumor. B. (L) long diameter of the lymph node; (S) short diameter of the lymph node. C. The mean CT value was calculated by delineating a region of interest that encompassed the entire lymph node, ensuring that the measurement line remained within the border of the lymph node. D. Reniform shape. E. Round shape. F. Other shape. G. Smooth border. H. Indistinct border. I. Fused border.

**Figure 2:**
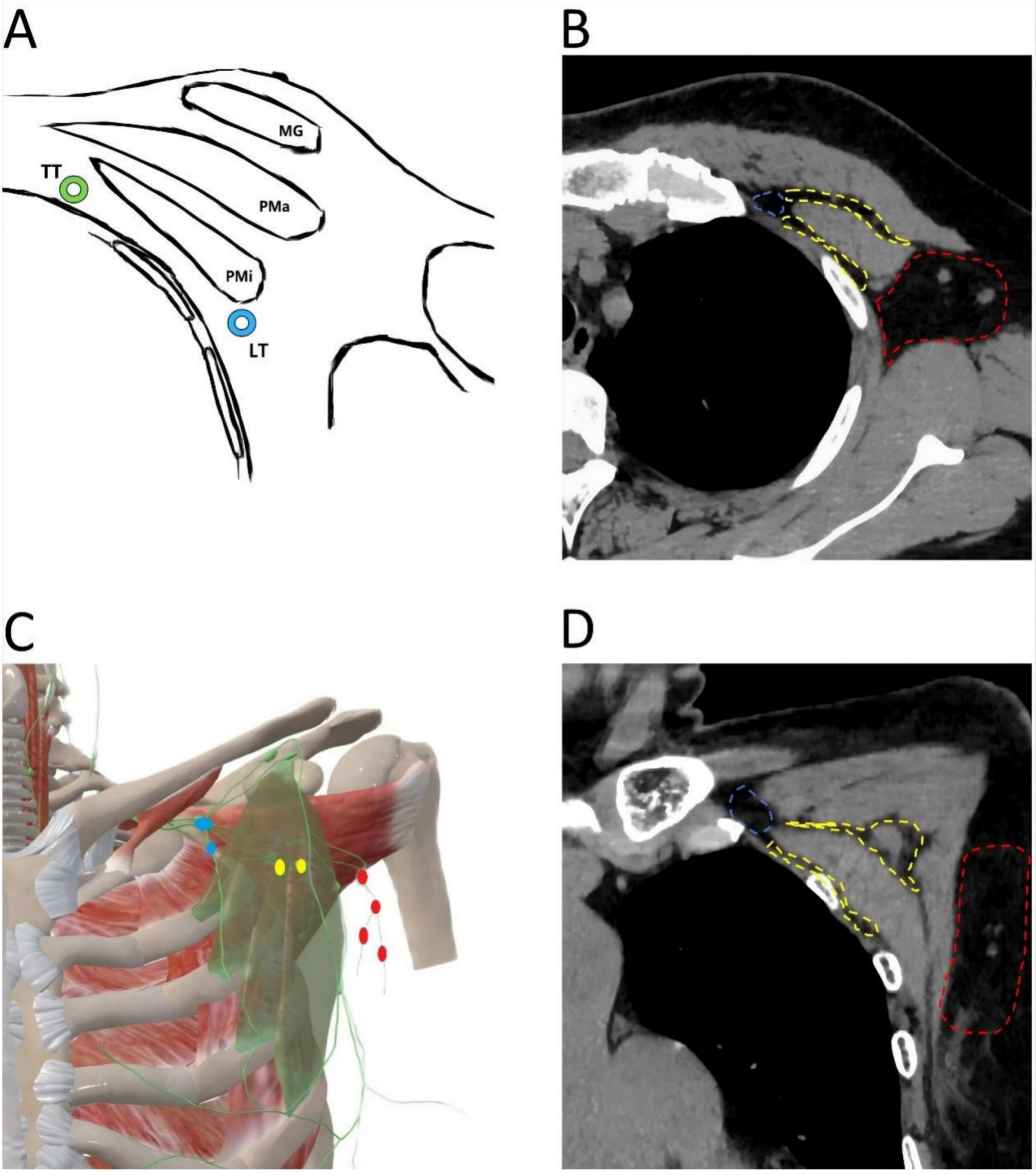
Anatomical location of axillary lymph node. A. Transverse anatomical schematic. MG: mammary gland, Pma: pectralis major muscle, Pmi: petcoralis minor muscle, LT: lateral thoracic artery (blue), TT: thoracic acromial artery (green). B. Anatomical location of axillary lymph node in a CT transverse view. The area where the level I axillary lymph node is located (red); The area where the level II axillary lymph node is located (yellow); The area where the level III axillary lymph node is located (blue). C. Coronal anatomical schematic. The pectoralis minor muscle (light green), level I axillary lymph node (red), level II axillary lymph node (yellow), level III axillary lymph node (blue). D. Anatomical location of axillary lymph node in a CT coronal view. The area where the level I axillary lymph node is located (red); The area where the level II axillary lymph node is located (yellow); The area where the level III axillary lymph node is located (blue).

**Figure 3:**
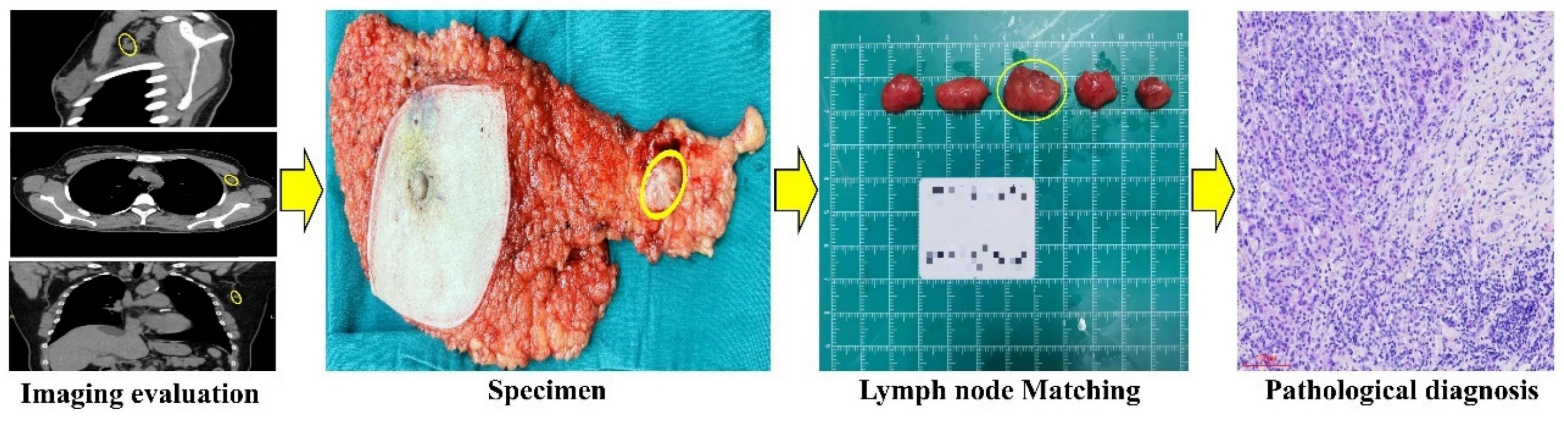
Flow chart comparing imaging to pathology. CT detected a 2x1.5cm lymph node located laterally to the left pectoralis minor. The node was surgically located, and subsequent pathology confirmed breast cancer metastasis.

The patient's basic information, HRCT imaging, and pathology (both the preoperative primary tumor pathology and the postoperative lymph node pathology) were recorded in accordance with data protection regulations. A radiologist with over 15 years of experience in breast imaging analyzed the HRCT images preoperatively, carefully selecting the region of interest (ROI) on the PACS while avoiding adjacent blood vessels and muscular tissues. The key parameters of the ALN were documented, including mean CT value, diameters, border, and shape (Figure 1). Moreover, the anatomical location of each ALN was recorded, utilizing Berg's localization criteria and referencing landmarks such as the pectoralis minor, adjacent vessels, and ribs. Anatomically, ALN are classified into three levels based on their relation to the pectoralis minor muscle. The origin of the thoracic acromial artery is located at the projected position of the inner edge of the pectoralis minor muscle, while the origin of the lateral thoracic artery is situated at the projected position of the outer edge of the pectoralis minor muscle. Level III (subclavian area) is situated medially to the inner edge of the pectoralis minor muscle and is aligned with the origin of the thoracic acromial artery. In contrast, Level I is situated lateral to the outer edge of the pectoralis minor and is marked by the starting point of the lateral thoracic artery. The area situated between the origins of the thoracic acromial and lateral thoracic arteries, which also encompasses the interpectoral (Rotter's) lymph nodes, is designated as Level II (Figure 2). All patients underwent sentinel lymph node biopsy or axillary lymph node dissection within one week of undergoing a chest HRCT examination. The fresh tissues were placed on a specimen measuring board and subsequently sent to the pathology department. A collaborative effort between pathologists, breast surgery experts, and radiology experts ensured that the specimens were matched and numbered individually based on the location and size of the lymph node corresponding to the HRCT imaging. All specimens obtained postoperatively underwent formalin fixation, paraffin embedding, and hematoxylin and eosin (HE) staining. Both micro- and macro-metastasis were considered MLN. Figure 3 depicts the complete process of comparison of imaging with pathology.

### Statistical analysis

All analyses were performed using R version 4.4.0 (http:// www.Rproject.org). The normality of the continuous variables was evaluated using the Kolmogorov-Smirnov test. Data that adhere to a normal distribution was presented as the mean ± standard deviation (SD). For intergroup comparisons, the independent sample t test was employed. In instances where the data did not adhere to a normal distribution, they were expressed as the median accompanied by interquartile ranges (IQRs). Comparative analyses between the two groups were conducted using the Mann-whitney U test. Categorical variables were compared between groups using the chi-square test. The LASSO regression, which is particularly suitable for reducing high-dimensional data, was employed to identify the most predictive risk factors among MLN. Subsequently, a multivariate logistic regression analysis was conducted using variables that had been screened by LASSO regression to construct a predictive model. A nomogram was developed based on the logistic regression model. The performance of the nomogram was evaluated using the concordance index (C-index) and calibration plot. The receiver operator characteristic (ROC) curve and the area under the curve (AUC) were used to assess the diagnostic value. The nomogram was subjected to bootstrapping validation (1000 bootstrap resamples) to calculate a relatively corrected C-index and AUC. Decision curve analysis was conducted to determine the clinical usefulness of the nomogram by quantifying the net benefits at different threshold probabilities in the breast cancer cohort. All statistical significance levels were two-sided, with a p-value threshold of <0.05.

## Results

### Patient characteristics

From April 2023 to May 2024, this study prospectively enrolled 98 cases. Based on whether the patients developed ALN metastasis of breast cancer, these cases were divided into two groups: the metastasis group and the non-metastasis group. The metastasis group comprised 30 cases, while the non-metastasis group consisted of 68 cases. Table 1 presents a summary and comparison of the basic characteristics of the two groups.

### Imaging features of axillary lymph node

**Figure 4:**
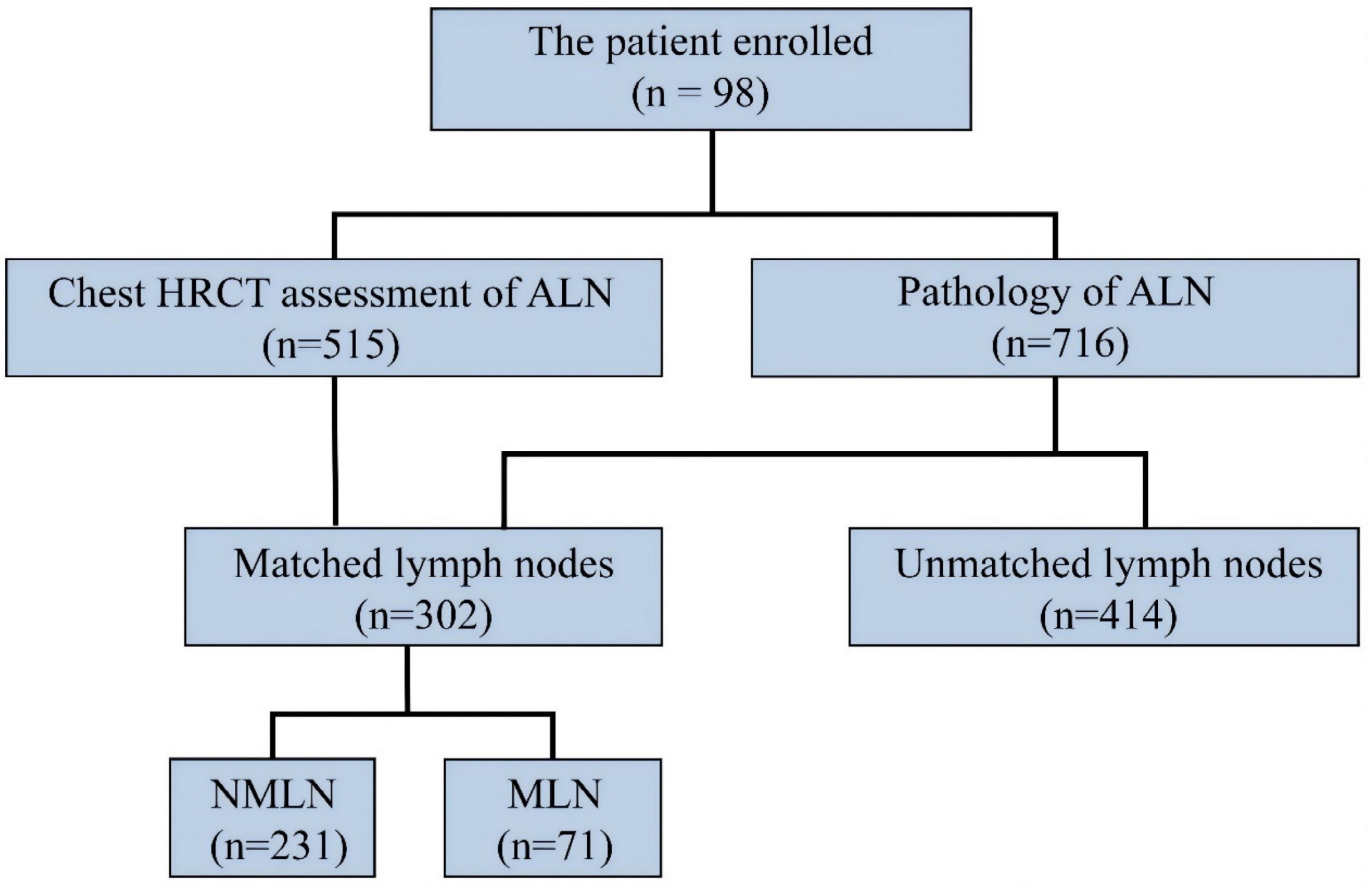
Imaging versus the pathology in 716 lymph node of 98 patients. Abbreviations: ALN-axillary lymph node; MLN-metastasis lymph node; NMLN-non-metastasis lymph node.

**Figure 5:**
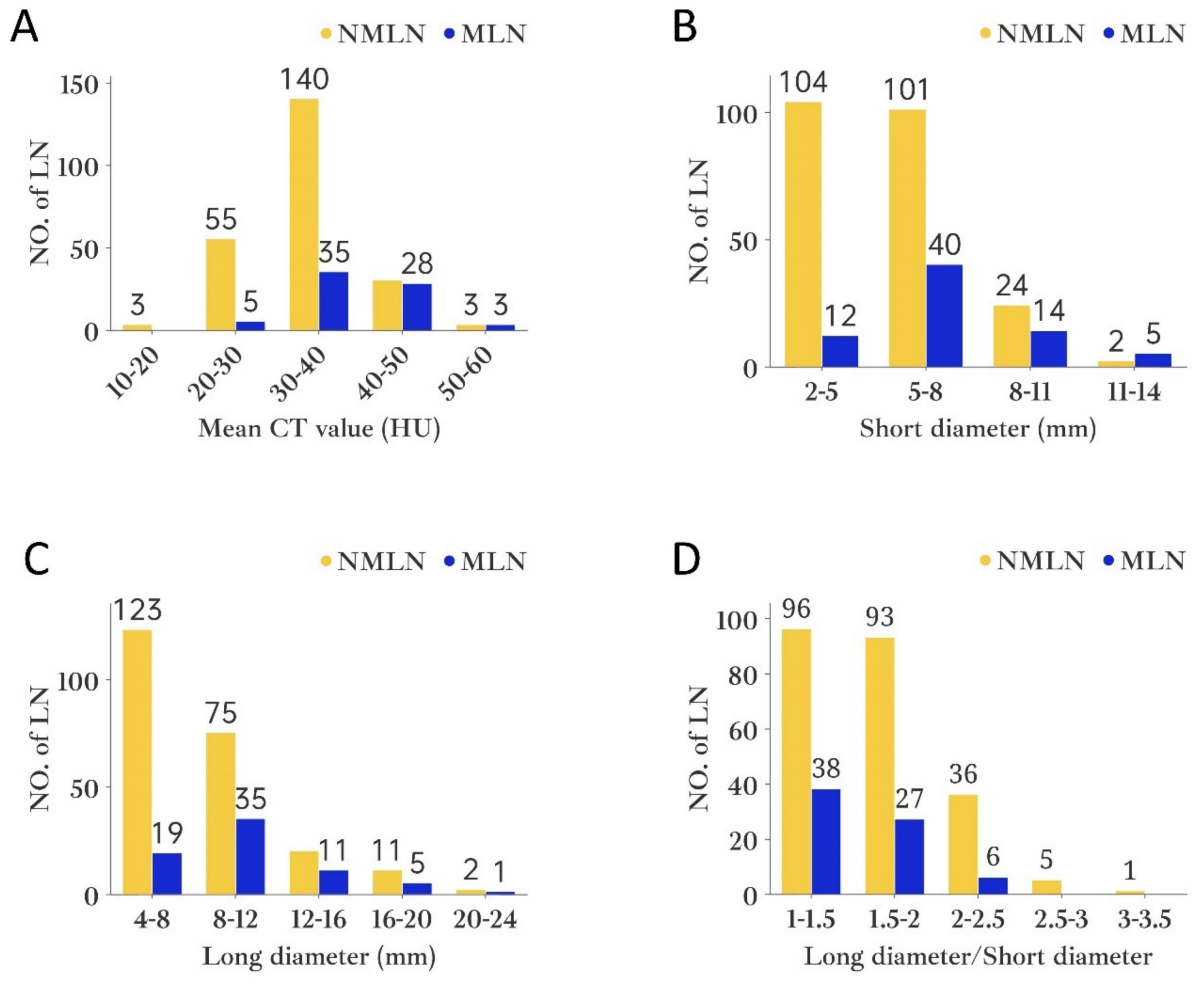
The histogram of axillary lymph node distribution under different variables. A. Mean CT value; B. Short diameter; C. Long diameter; D. Long diameter/Short diameter. Abbreviations: NO.of LN- number of lymph nodes.

For the purpose of the node-by-node assessment, only those ALN that could be identified were considered. HRCT identified 515 ALN, whereas pathological examination revealed 716. It is noteworthy that 302 ALN were successfully matched by both HRCT imaging and pathology. Among them, pathology confirmed 71 as MLN and 231 as NMLN (Figure 4). The mean CT value, short and long diameter of MLN significantly exceeded those of NMLN (both P<0.001). However, MLN exhibited a lower long diameter/short diameter (L/S) ratio compared to NMLN (P=0.003). The median short diameter of MLN was 6.1mm (IQRs: 3.3mm; range: 3.1-13mm), while that of NMLN was 5.0mm (IQRs: 1.8mm; range: 2-13.8mm). Similarly, the median long diameter of MLN was 8.9mm (IQRs: 4.1mm; range: 5.5-20mm) compared to 7.8mm (IQRs: 3mm; range: 4.4-21.6mm) for NMLN. The median L/S ratio for MLN was 1.45 (IQRs: 0.2; range: 1.06-2.40), whereas for NMLN, it was 1.61 (IQRs: 0.51; range: 1.03-3.20). Finally, the mean CT value for MLN was 38.99 HU (SD: 6.80HU), and for NMLN, it was 33.05 HU (SD: 6.32HU). Figure 5 depicts the histogram of lymph nodes distribution under different diameters, L/S ratio, and mean CT value. Notably, significant differences in border and shape were found between MLN and BLN (P=0.003 and P<0.001, respectively) (Table 2).

### Feature selection

**Figure 6:**
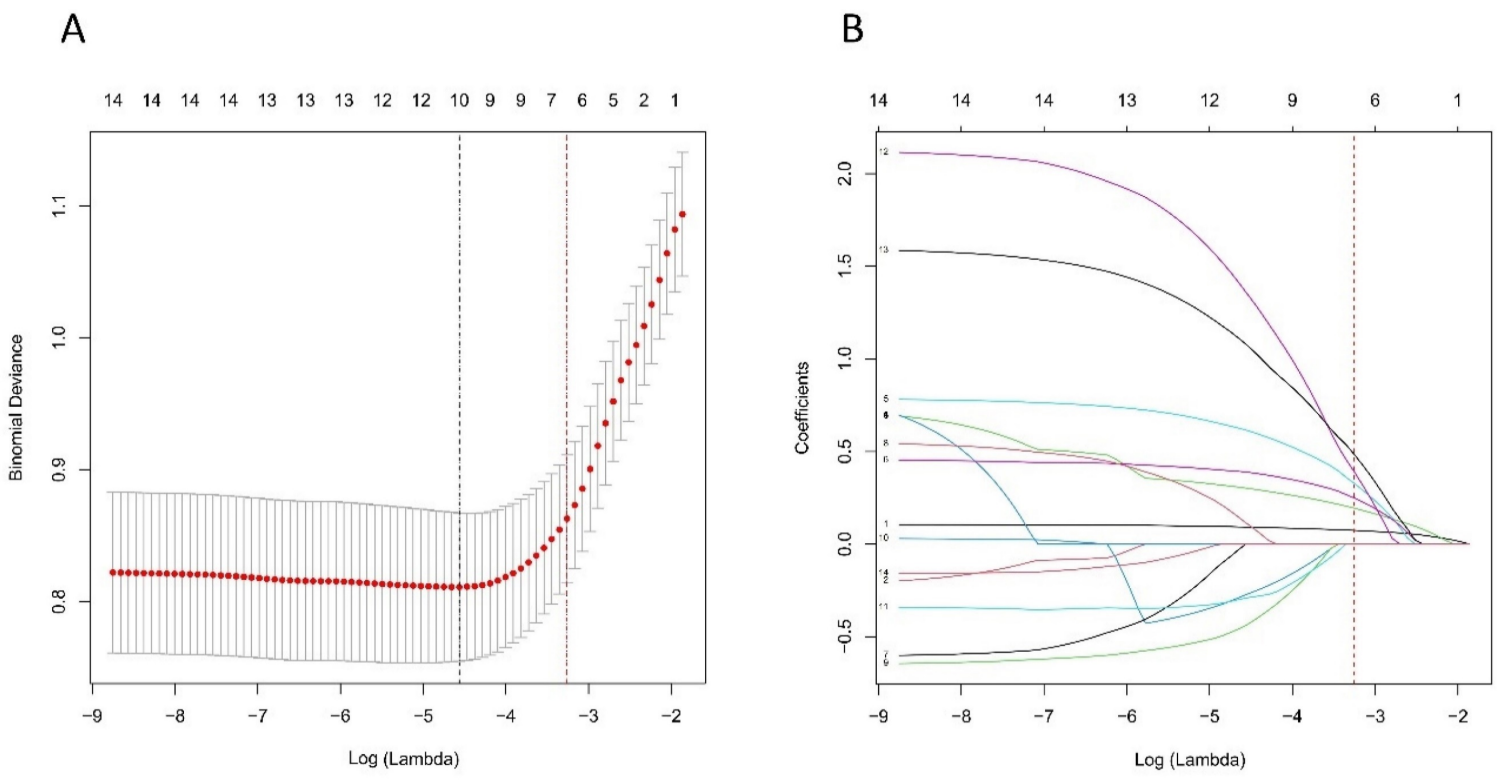
Selection of HRCT imaging and pathology features using the LASSO regression. A. Optimal lambda selection in the LASSO model involved fivefold cross-validation based on minimum criteria. The binomial deviance curve was plotted against log(lambda), with dotted lines indicating optimal values determined by both the minimum criteria and the 1-SE criteria (red). B. The LASSO coefficient profiles for 14 features were plotted versus log (lambda). A vertical line (red) indicates the optimal lambda chosen through fivefold cross-validation, resulting in six features with nonzero coefficients. Abbreviations: HRCT-high-resolution computed tomography; LASSO-least absolute shrinkage and selection operator; SE-standard error.

Based on the cohort comprising 98 patients with 302 lymph nodes, we initially identified 14 features spanning demographics, HRCT imaging, and pathology. As illustrated in Figure 6A and B, through the application of the LASSO regression, these features were further refined to identify 10 potential predictors. By leveraging the 1 Standard Error (SE) of the minimum criteria, we successfully streamlined the model to encompass six strongly correlated variables: mean CT value, short diameter, border, shape, Ki-67 status, and histological grade. This streamlining not only mitigates the risk of overfitting but also enhances computational efficiency, thereby bolstering the model's robustness and overall performance.

### Development of an individualized prediction model

**Figure 7:**
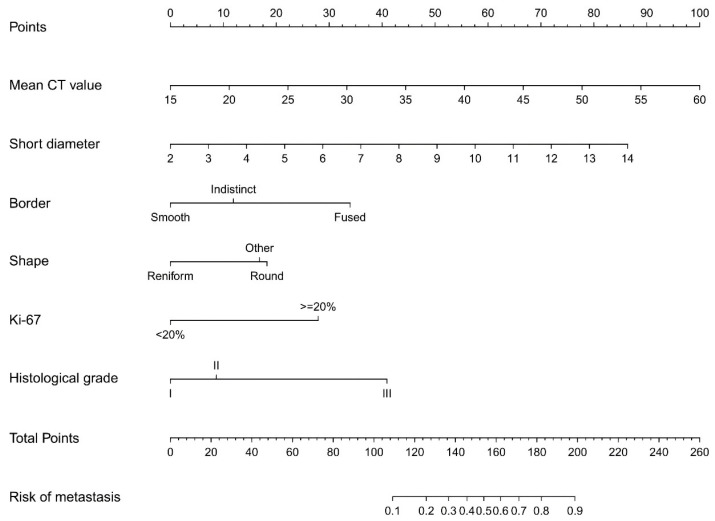
Developed prediction nomogram. The axillary lymph node metastasis nomogram was developed in the cohort, with the mean CT value, short diameter, border, shape, Ki-67 status, and the histological grade.

The results of the logistic regression analysis among the mean CT value, short diameter, border, shape, Ki-67 status, and histological grade are given in Table 3. The model that incorporated the above independent predictors was developed and presented as the nomogram (Figure 7).

### Performance of the nomogram

**Figure 8:**
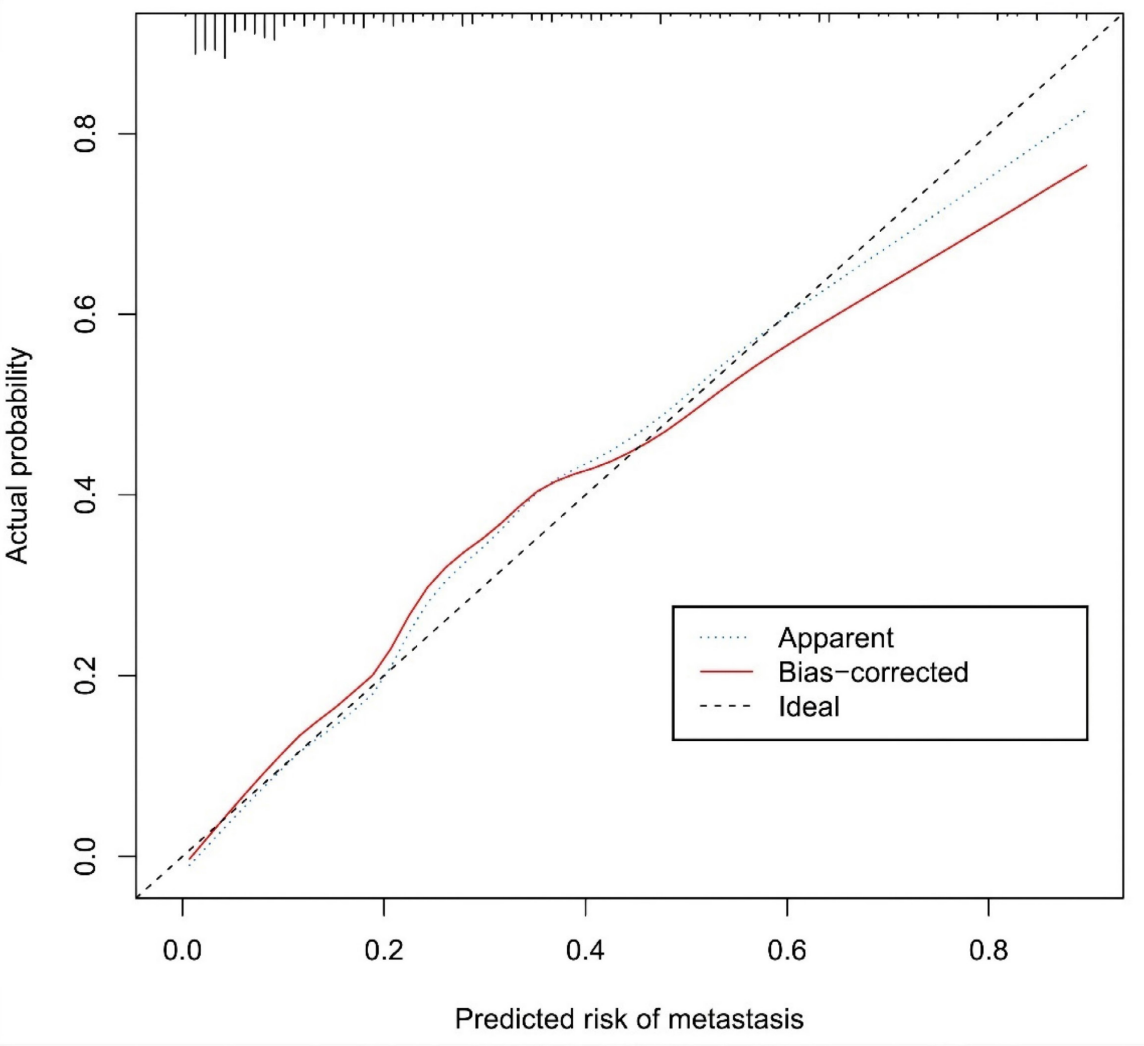
Calibration curves of the prediction nomogram in the cohort. The x-axis represents the predicted axillary lymph node metastasis risk. The y-axis represents the actual diagnosed axillary lymph node metastasis. The diagonal dotted line represents a perfect prediction by an ideal model. The blue dotted line represents the entire cohort, and the red solid line is bias-corrected by bootstrapping (B=1000 repetitions, boot), indicating nomogram performance.

**Figure 9:**
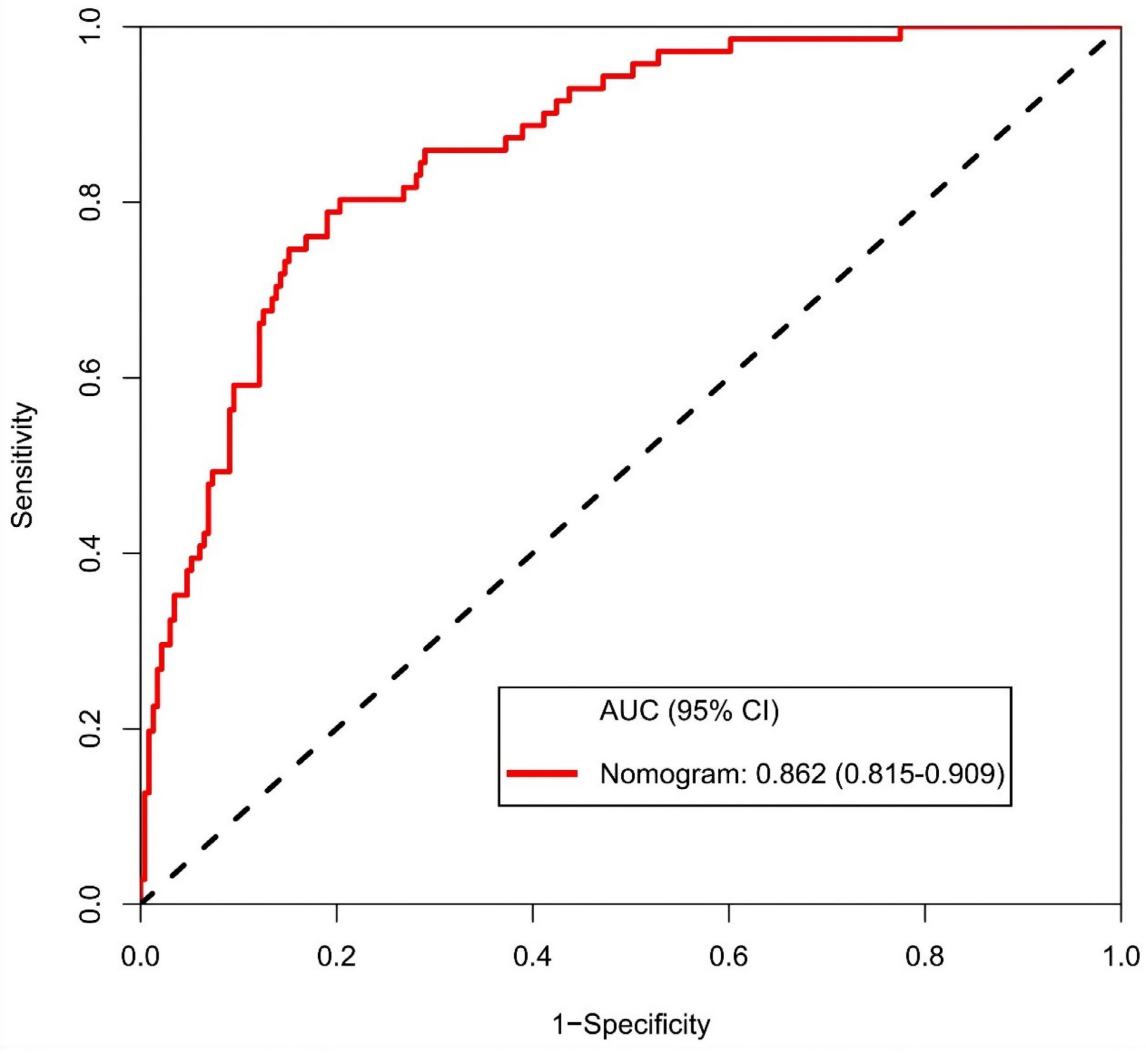
Receiver operating characteristic curve and prediction model based on nomogram. Abbreviations: AUC-area under the curve; CI-confidence interval.

**Figure 10:**
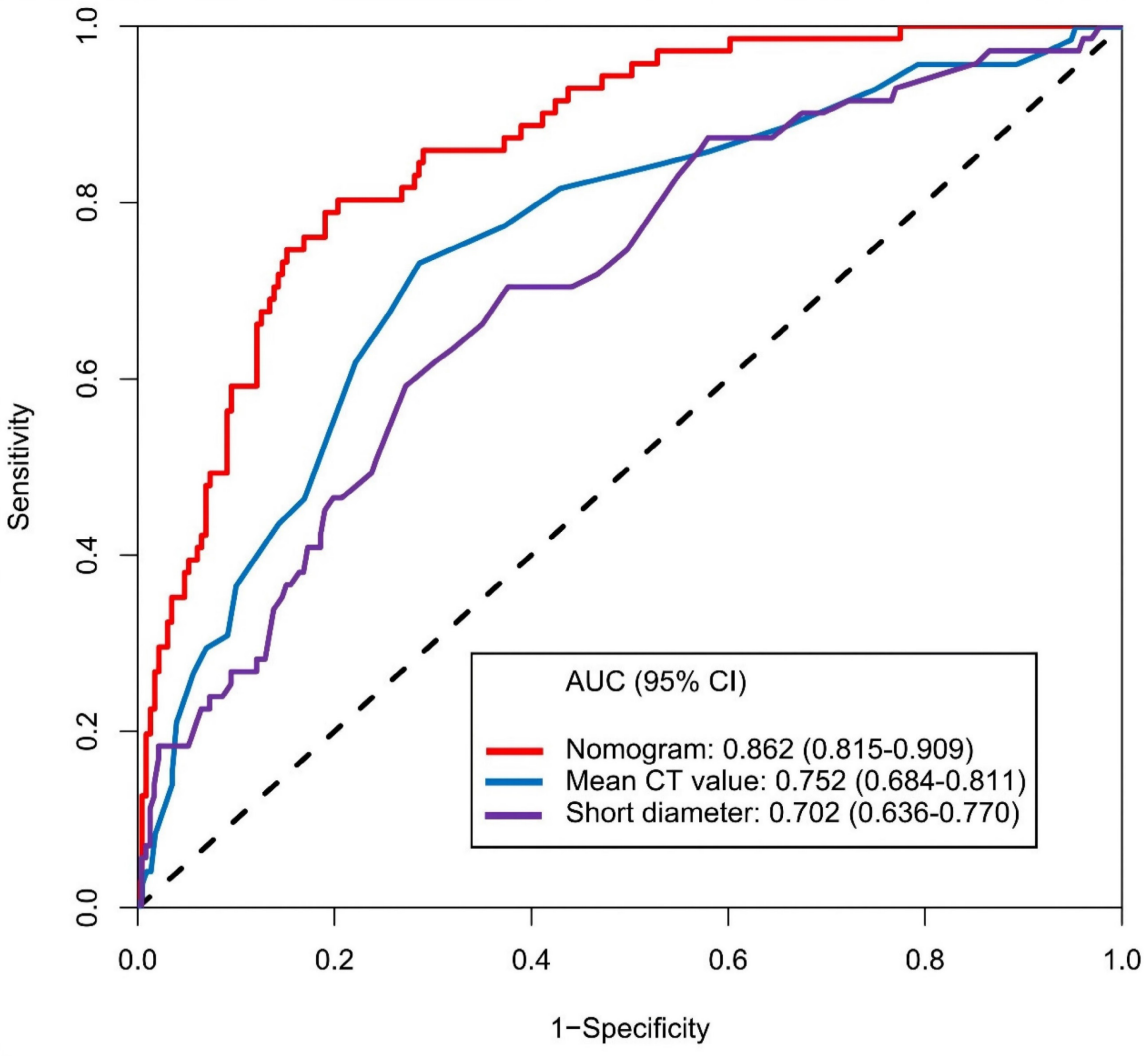
Receiver operating characteristic curve and prediction model based on mean CT value, short diameter and nomogram. Abbreviations: AUC-area under the curve; CI-confidence interval.

The calibration curve of the nomogram for the prediction of ALN metastasis in breast cancer patients demonstrated good agreement in this cohort (Figure 8). The prediction nomogram for the cohort achieved a C-index of 0.869 (95% CI: 0.826-0.912), with bootstrapping validation confirming a score of 0.845. Likewise, the nomogram showed a diagnostic accuracy of 79.5%, an AUC of 0.862 (95% CI: 0.815-0.909), sensitivity of 78.9%, and specificity of 79.7% (Figure 9). Additional bootstrapping validation further supported an AUC of 0.857. Furthermore, the AUC determined using the mean CT value was 0.752 (95% CI, 0.684-0.811), while the AUC determined using the short diameter was 0.705 (95% CI, 0.636-0.770) (Figure 10). Taken together, these findings underscore the model's excellent discriminatory and predictive abilities.

### Clinical use

**Figure 11:**
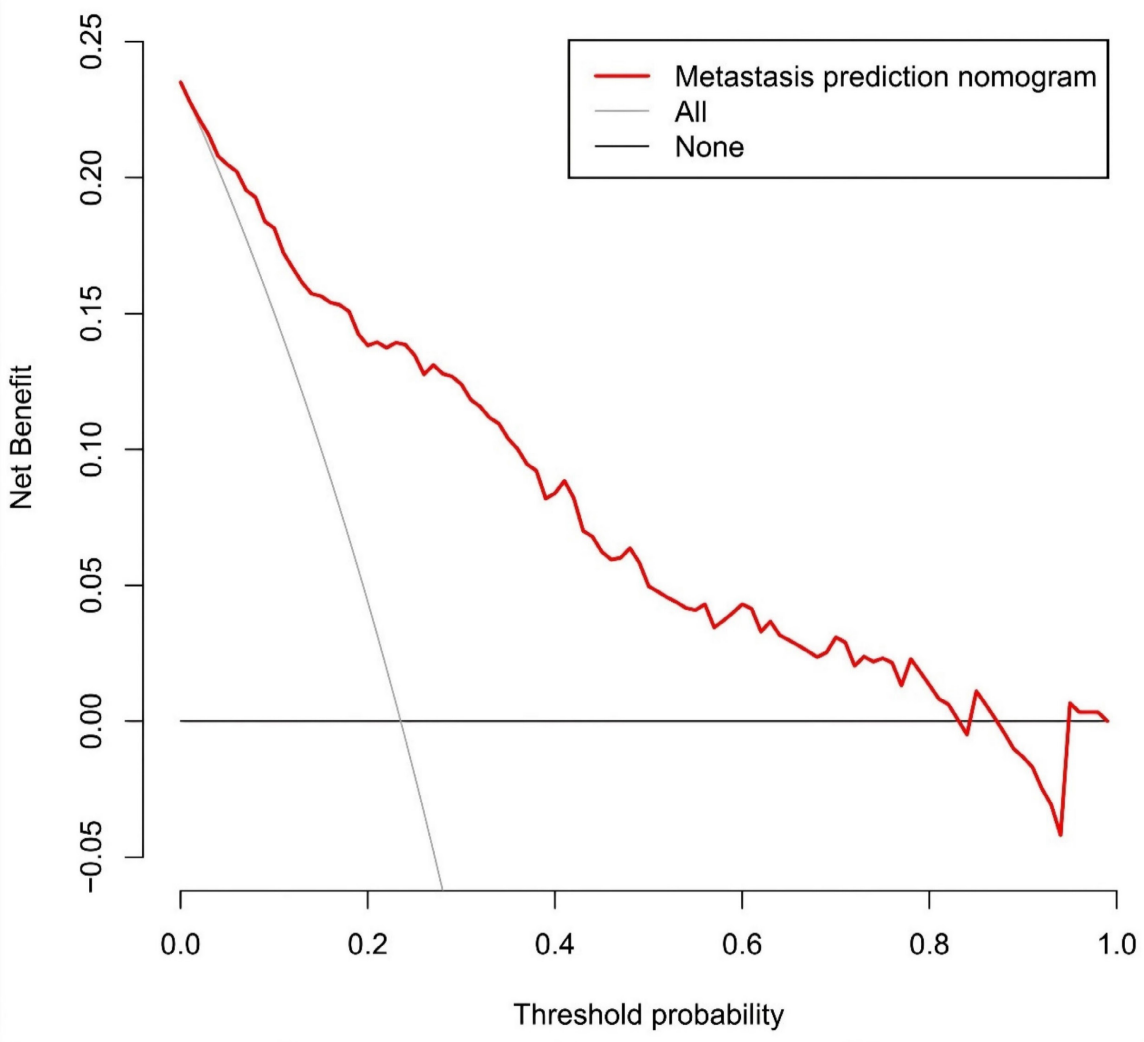
Decision curve analysis for the prediction nomogram. The y-axis measures the net benefit. The red line represents the axillary lymph node metastasis risk nomogram. The thin solid line represents the assumption that all axillary lymph node are malignant. The thick solid line represents the assumption that no axillary lymph node are malignant.

Figure 11 presents the decision curve analysis for the prediction nomogram. The curve demonstrates that utilizing this nomogram to predict the risk of MLN provides greater benefit than the scheme, particularly when the threshold probability of MLN ranges from 1 to 86%. Within this range, net benefit was comparable with several overlaps, on the basis of the metastasis nomogram.

## Discussion

In clinical practice, preoperative non-invasive precise assessment of lymph node metastasis and accurate localization in breast cancer patients is an urgent issue that needs to be addressed. Preoperative imaging radiology is undoubtedly a very good tool, but it also presents significant challenges^16^. CT technology appeared in the 1970s but was rarely used for breast diseases^17^-^19^. However, in the 1990s, advanced spiral CT scanners improved imaging without extra radiation, aiding in assessing ALN status^20^,^21^. The integration of spiral CT scanners with HRCT technology offers the potential to elucidate the subtle structural and morphological alterations of lymph nodes, thereby improving the precision of ALN status assessment. In this study, 31% (71/231) of the ALN were identified as MLN. In the risk factor analysis, six characteristics were found to be associated with MLN, including the mean CT value, short diameter, border, and shape of the ALN, as well as the Ki-67 status and histological grade of the primary tumor. The prediction nomogram indicated that higher mean CT value, larger short diameter, fused border, round shape, Ki-67≥20%, and histological grade III may be the key individual factors associated with MLN.

Hounsfield units (HU) serve as a quantifiable measure of X-ray attenuation in CT imaging, offering an objective and reproducible metric that is less prone to subjective examiner interpretation^22^,^23^. It is generally believed that pathological parameters, including cell count, tumor stroma, and/or extracellular matrix, may have a correlation with CT value^24^,^25^. As previous study indicated that HU can accurately distinguished breast masses as cystic or solid with or without contrast with chest CT^26^. Urata *et al.* conducted a retrospective, dual-center study on breast cancer patients who underwent contrast-enhanced CT assessment. They discovered that CT value can predict ALN status with sensitivity, specificity, and accuracy levels of 79.6%, 80.5% and 80.2%, respectively^27^. Similarly, Zhang *et al.*'s research indicated that the venous phase slope of the spectral HU curve is the optimal single parameter for detecting metastatic sentinel lymph nodes^28^. In our study, the nomogram that we established indicated that the mean CT value carried the greatest weight, implying that lymph nodes with a higher mean CT value have a higher likelihood of metastasis (Figure 7). Furthermore, the AUC, determined using mean CT value, stood at 0.752 (95% CI, 0.684-0.811). The optimal cut-off value was identified as 41.2 HU, which yielded a sensitivity of 73.2%, a specificity of 71.4%, and an accuracy of 71.9% (Figure 10). Assessing lymph node status by CT value is advantageous due to its objectivity and availability during preoperative exams. However, variations in CT scanners, imaging parameters, and software across institutions can affect results. Thus, when predicting ALN status with CT value, the cut-off may only be accurate for patients examined at the same institution.

In clinical practice, the size of lymph nodes serves as a critical indicator of their status^29^. From a pathological perspective, MLN enlarge due to the infiltration of tumor cells and obstructed lymphatic drainage^30^. Numerous imaging studies have also demonstrated that the diameters of MLN are significantly larger than those of NMLN^31^-^33^. This study further supported these observations. Our findings showed that the median short diameter of MLN was 6.1mm (IQRs: 3.3mm), while that of NMLN was only 5.0mm (IQRs: 1.8mm) (P<0.001). Similarly, the median long diameter of MLN is 8.9mm (IQRs: 4.1mm), which is significantly higher than the 7.8mm (3.0mm) of NMLN (P<0.001). Lymph node diameters may vary in imaging due to angle changes relative to the imaging plane. Compared to the long diameter, the short diameter offers a more stable, accurate representation of the size and higher measurement repeatability, as it is less affected by angle changes^34^-^36^. The findings of this study revealed that the long diameter is not an independent predictor of ALN status. Instead, the short diameter proved superior to the long diameter in anticipating ALN status (AUC: 0.702 vs. 0.637). The established nomogram showed that the short diameter was included with a relatively high weight, which means that ALN with larger short diameters are more likely to metastasize. These results align with the Response Evaluation Criteria in Solid Tumors 1.1, which recommends using the short diameter to distinguish lymph nodes status^37^.

However, there is still no consensus on the optimal cut-off value for the short diameter of MLN, which varies from 5mm to 10.4mm across different tumor types 12,29,38-42. Despite data differences among study groups, the diagnostic trend was uniform: a larger short diameter of lymph nodes led to higher positive predictive value and specificity in metastasis diagnosis, but lower negative predictive value and sensitivity. In our study, the optimal cut-off value for the short diameter in predicting MLN was found to be 7.4mm, yielding a sensitivity of 70.4%, a specificity of 62.3%, and an accuracy of 64.2% (Figure 10). Furthermore, studies have demonstrated a correlation between lymph nodes (including ALN) with a L/S ratio less than 1.5 and the presence of metastasis^43^-^45^. In this study, we found that the L/S ratio of MLN was significantly lower than that of BLN, with 53.5% (38/71) of MLN exhibiting an L/S ratio below 1.5. It is noteworthy that while univariate analyses revealed significant differences between long diameter and L/S ratio, LASSO regression indicated that these two factors exhibited diminished significance when considered alongside other variables. This finding was further corroborated by subsequent multifactor logistic regression analysis. This indicated that the independent effects of long diameter and L/S ratio on the results are diminished when interactions between multiple variables are considered. They may be masked by other stronger influences or covariates that interact with other factors.

Besides CT value and size, the border and shape of lymph nodes are crucial for assessing lymph node status^46^. The NMLN typically exhibit a reniform shape, smooth border, an absence of focal prominence, and a clearly visible central fatty hilum^47^,^48^. The immune response triggered by cancer cells, as well as the process of their colonization and proliferation, may result in significant morphological and border changes in ALN^49^. With its high spatial resolution, HRCT is capable of accurately capturing these subtle changes, which in turn provides substantial support in assessing the status of the lymph nodes. Similar to previous studies, we found that MLN exhibited an odds ratio (OR) of 5.296 (95% CI: 1.618-17.919) for fused border and an OR of 2.45 (95% CI: 1.137-5.371) for a round shape in comparison to NMLN. Different from previous studies, indistinct border and other shape (such as lobulated or focally prominent) may not necessarily indicate MLN. Perhaps the border and shape of ALN are also affected by various factors, including non-neoplastic inflammation and fat content^50^-^54^. To optimize diagnostic accuracy, considering multiple factors like tumor metabolic intensity and inflammatory indicators is imperative.

Scholars believe that correlating the biological behavior of lymph node metastasis with the pathological characteristics of the primary tumor can not only improve the accuracy of judging lymph node metastasis but also provide clinical support for finding new targets for breast cancer treatment^55^,^56^. Previous studies have discovered a strong association between larger primary tumor diameter, vascular invasion, triple-negative breast cancer, and MLN^57^-^60^. However, most of these risk factors were based on postoperative pathological results, making them potentially unsuitable for the preoperative evaluation of ALN status. In our study, all histological features of the primary tumor were derived from preoperative CNB^61^-^63^. High histological grade (grade III) and Ki-67≥20% of the primary tumor were identified as independent predictors of MLN, both of which carried relatively high weights in the nomogram. However, characteristics such as HR, HER-2 and tumor diameter did not show significant differences. This finding may be due in part to the lack of HER2 information for some patients in this study (11/98), as well as the failure of CT scans to detect the primary tumor (16/98).

To the best of our knowledge, this is the first prospective study to create a nomogram using preoperative pathology and HRCT imaging for accurate preoperative ALN status evaluation. We developed and validated this prediction nomogram using six accessible variables to assess the status of each ALN in patients with breast cancer. The nomogram boasts user-friendly digital interface, enhanced accuracy, and prognostic reports that are simpler to comprehend, all aimed at facilitating improved clinical decision-making.

### Limitations

The present study was limited by several factors. Primarily, despite extensive internal validation via bootstrap testing, our nomogram lacks external validation. Its applicability to other breast cancer populations remains uncertain, necessitating further external evaluation in broader contexts. Secondly, establishing a one-to-one correspondence between excised lymph nodes and HRCT imaging of ALN proved challenging, resulting in a relatively small number of matched ALN, particularly MLN. Thirdly, despite efforts to match all histologically identified lymph nodes, there was a notable discrepancy in the number of nodes within different short diameters, which could potentially impact the accuracy of the results.

## Conclusions

This study provided a relatively accurate prediction tool of ALN status for breast cancer patients. However, these results should be interpreted cautiously as further extensive prospective studies are required to validate the diagnostic efficacy of the nomogram.

## Figures and Tables

**Table 1:** Comparison of characteristics between the metastasis and non-metastasis group.

Characteristic	Metastasis group N, %	Non-metastasis group N, %	Total N, %
Age (years)			
≤40	4 (13.3)	7 (10.3)	11 (11.2)
>40	26 (86.7)	61 (89.7)	87 (88.8)
Menstrual State			
Premenopausal	11 (36.7)	23 (33.8)	34 (34.7)
Postmenopausal	19 (63.3)	45 (66.2)	64 (65.3)
BMI			
<18.5	2 (6.7)	5 (7.4)	7 (7.1)
18.5-24	16 53.3)	43 (63.2)	59 (60.2)
>24	12 (40)	20 (29.4)	32 (32.7)
Tumor diameter (cm)			
≤2	19 (63.3)	46 (67.6)	65 (66.3)
>2	6 (20)	11 (16.2)	17 (17.3)
Unknown	5 (16.7)	11 (16.2)	16 (16.4)
Histological grade			
I	2 (6.7)	7 (10.3)	9 (9.2)
II	12 (40)	44 (64.7)	56 (57.1)
III	16 (53.3)	17 (25)	33 (33.7)
Hormone receptor			
Negative	10 (33.3)	16 (23.5)	26 (26.5)
Positive	20 (66.7)	52 (76.5)	72 (73.5)
HER-2			
Negative	23 (76.7)	40 (58.8)	63 (64.3)
Positive	4 (13.3)	20 (29.4)	24 (24.5)
Unknown	3 (10)	8 (11.8)	11 (11.2)
Ki-67 status			
<20%	6 (20)	30 (44.1)	36 (36.7)
≥20%	24 (80)	38 (55.9)	62 (63.3)

Abbreviations: BMI-body mass index; Unknown-the information is unclear.

**Table 2:** HRCT imaging of axillary lymph node

Feature	MLN (n=71) N,%	NMLN (n=231) N,%	P value
Short diameter (mm), median (IQRs)	6.1 (3.3)	5.0 (1.8)	<0.001**
Long diameter (mm), median (IQRs)	8.9 (4.1)	7.8 (3)	<0.001**
L/S Ratio	1.45 (0.2)	1.61 (0.51)	0.003**
Mean CT value (HU), mean ± SD	38.99±6.80	33.05±6.32	<0.001***
Border			0.003*
Smooth	46 (64.8)	187 (81)	
Indistinct	14 (19.7)	34 (14.7)	
Fused	11 (15.5)	10 (4.3)	
Shape			<0.001*
Reniform	24 (33.8)	143 (61.9)	
Round	29 (40.8)	60 (26)	
Other	18 (25.4)	28 (12.1)	

Abbreviations: *chi-square test; **Mann-whitney U test; ***independent sample t test.

**Table 3:** Logistic multivariate analysis

Variable	β	Standard.Error	Z	Wald χ2	Odds ratio (95% CI)	P-value
Mean CT value	0.1091	0.0265	4.1173	16.9525	1.115 (1.061-1.177)	<0.001
Short diameter	0.3533	0.0865	4.0858	16.6935	1.424 (1.207-1.697)	<0.001
Border						
Smooth	Reference					
Indistinct	0.5848	0.4492	1.302	1.6951	1.795 (0.734-4.317)	0.193
Fused	1.6669	0.6073	2.7446	7.5329	5.296 (1.618-17.919)	0.006
Shape						
Reniform	Reference					
Round	0.8959	0.3941	2.2734	5.1684	2.45 (1.137-5.371)	0.023
Other	0.8257	0.4533	1.8214	3.3176	2.284 (0.932-5.563)	0.069
Ki-67 status						
Ki-67<20%	Reference					
Ki-67≥20%	1.3702	0.413	3.3178	11.0077	3.936 (1.800-9.148)	<0.001
Histological grade						
I	Reference					
II	0.4264	0.6011	0.7094	0.5033	1.532 (0.492-5.310)	0.478
III	2.0086	0.6341	3.1679	10.0353	7.453 (2.269-27.757)	0.002
